# Editorial: XVII Spanish Portuguese Congress on Plant Biology (BP2021) - plant biochemistry and metabolism

**DOI:** 10.3389/fpls.2023.1189060

**Published:** 2023-04-14

**Authors:** Rogelio Santiago, José A. Monreal

**Affiliations:** ^1^ Misión Biológica de Galicia, Spanish Council for Scientific Research (MBG-CSIC), Pontevedra, Spain; ^2^ Agrobiología Ambiental, Calidad de Suelos y Plantas Universidade de Vigo (UVIGO), Unidad Asociada a la MBG Consejo Superior de Investigaciones Científicas (CSIC), Vigo, Spain; ^3^ Departamento de Biología Vegetal y Ecología, Facultad de Biología, Universidad de Sevilla, Sevilla, Spain

**Keywords:** plant biochemistry, plant metabolism, secondary metabolites, abiotic stress, biotic stress

Between the 7^th^ and the 9^th^ of July 2021, the Spanish Society of Plant Biology and the Portuguese Society of Plant Physiology celebrated the “XVII Spanish-Portuguese Congress on Plant Biology (BP2021)”, online on this occasion because of COVID-19 restrictions. The congress was organized in 12 scientific sessions covering matters related to plant biology, from basic aspects to more applied regards. Participants interested were invited to send their communications to one of the 4 Research Topics launched: Plant Biochemistry and Metabolism, Plant Growth and Development, Plant Responses to Environmental Stress, or Gene Expression and Genetic Modification of Plants. In the topic Plant Biochemistry and Metabolism, 6 original research papers and 1 review were accepted for publication. Plant biochemistry, and its relation with plant metabolism, is a very wide field in Plant Science with important consequences for plant growth, productivity, or defence against biotic and abiotic stresses.

In relation to specialized metabolites, we cover topics related to their role in response to the environment, and the regulation and time of the day production.

A large variety of secondary metabolites have been identified in plants, and many of those compounds exhibit strong allelopathic activities inhibiting seed germination and growth of other plants (Macías et al., 2019). Finding crop germplasm resources with allelopathic activity and identifying related phytotoxic metabolites may be helpful in developing weed management options for sustainable agriculture. Previous studies have demonstrated significant allelopathic activity of sweet potato on various plants (Shen et al., 2018). However, the metabolites of sweet potato responsible have not been extensively studied. In the paper by Shen et al. the authors define the chemical composition of petroleum ether extracts of sweet potato in relation to the allelopathic effects on four invasive plant species; concluding that linoleic acid, palmitic acid and ethyl palmitate represent the most promising allelochemicals for weeds control.

On the other hand, salinity is an abiotic stress that hampers plant physiological functioning in a number of ways, resulting in lower crop production. One of the strategies to mitigate salt stress in sustainable agriculture is the use of natural plant extracts instead of chemical fertilizers. Plant extracts fall under the umbrella of plant biostimulants, and are used to enhance plant growth (Drobek et al., 2019). In a complete and interesting review, Ahmad et al. define how plant extracts have been reported to enhance salt tolerance in plants, primarily through modulation of signaling and pathways, and the regulation of redox machinery. Authors also highlights the role of plant extracts against salinity in terms of their sources, methods of preparation, and mode of action.

Attending to the commercial production for pharmaceutical purposes of specialized metabolites Escrich et al. and Perez-Matas et al. provide stimulating insights in the control and regulation of paclitaxel production in *Taxus* spp. cell cultures. One of the limitations in taxanes production (paclitaxel, the active principle of Taxol^®^) is the gradual loss of specialized metabolite production during *in vitro* culture maintenance, which represents an important barrier in the development of large-scale production systems (Beum et al., 2004). Higher DNA methylation levels have been correlated with a low yield of specialized metabolites *in vitro* plant cell cultures (Sanchez-Muñoz et al., 2019). Therefore, Escrich et al. compared a low producer cell line (of 14 years old) and a new cell culture recently induced from fresh plant material to reveal specific methylation changes during its maintenance in optimal conditions. The promoter region of three taxane biosynthetic genes was studied, and a different pattern attending to the methylation accumulation was described, providing novel insights for designing strategies for the improvement of paclitaxel production. Furthermore, Perez-Matas et al. studied the taxane production in cell cultures treated with the elicitor coronatine or coronatine plus methyl-b-cyclodextrins, and compared with untreated control cultures. In addition, due to the recently described role of lipid droplets in the storage and trafficking of lipid compounds in cells, the characteristics and taxane accumulation capacity of these organelles was studied. After a comprehensive discussion authors conclude that elicitation with coronatine is an efficient strategy to increase paclitaxel production in cell cultures, which can be further enhanced by co-supplementation with cyclodextrins to promote the release of paclitaxel from the producer cells. The importance of the lipid droplets in the accumulation and trafficking of paclitaxel was also demonstrated, as these organelles increased in number after the elicitation of cell cultures, and the anticancer agent was the main taxane stored inside.

Lastly, regarding the timing of production of defensive metabolites, Doghri et al. analysed the accumulation of metabolites produced by 2 Brassica species (*Brassica oleracea* and *Brassica rapa*) following mechanical wounding at different times of the day: dawn (Z0), mid-day (Z4), and dusk (Z8). They found glucosinolates (GSLs) being differentially accumulated depending on the time of wounding, indicating a role for the circadian clock in this accumulation. Interestingly, the maximal accumulation of GSLs varied considerably depending of the specie. Moreover, authors carried out feeding experiments using the generalist pest *Mamestra brassicae* on brassica leaf disks previously primed by mechanical wounding. In a metabolome analysis, authors found chemical defence metabolites against larvae of *M. brassicae* being accumulated differentially depending on the time of wounding, with a maximal accumulation at Z0 for *B. oleracea* and ZT8 for *B. rapa*. This accumulation had important consequences on larvae feeding behaviour. These results show the importance of circadian rhythms in plant defence responses, and have important implications in the study of plant-herbivore interactions.

In relation to plant growth and development, steryl esters stored in cytoplasmic lipid droplets are the subject of the research paper presented by Burciaga-Monge and collaborators. Steryl esters serve as a reservoir of free sterols needed to meet the high demand of these plasma membrane components during rapid plant organ growth and expansion. The esters formation implies the esterification of a long-chain fatty acid group catalyzed by a family of enzymes known as sterol acyltransferases, which can be classified into acyl-CoA:sterol acyltransferases (ASAT) and phospholipid:sterol acyltransferases (PSAT). In a previous work, authors cloned and characterized the SlPSAT1 and SlASAT1 enzymes of tomato (Lara et al., 2018). This allowed undertaking the study reported here, in which a tomato CRISPR-Cas9-edited loss-of-function mutans lacking PSAT1 and ASAT1 were characterized. Altogether, results indicate that SlPSAT1 has a predominant role in tomato steryl esters biosynthesis, while SlASAT1 would mainly regulate the flux of the sterol pathway. This work contribute to expand the current understanding on the role of steryl esters and the enzymes involved in their synthesis in tomato germination, early development and senescence.

Finally, Peinado-Torrubia et al. deepen in the benefits of the chloride nutrition in the nitrogen assimilation and photorespiration. Since plants only use 30-40% of N applied to soils, a greater N use efficiency (NUE) is required to improve the sustainability, yield and quality of crops (Bijay and Craswell, 2021). In this work, authors assessed the unknown effect of beneficial Cl^−^ nutrition on NO^−3^ allocation and N metabolism. Results demonstrate for the first time that chloride improves NUE by promoting NO_3_^-^ assimilation through the simultaneous stimulation of both NO_3_^-^ reduction and NH_4_^+^ production from the photorespiration pathway, without altering the NO_3_^-^ uptake and transport. Therefore, Cl^−^ must be considered a good candidate to reduce both N application in farmlands and deleterious NO_3_^-^ content in vegetables. A complete integrative model of anatomical, cellular and metabolic responses to chloride nutrition at macronutrient levels is presented and discussed.

Overall, the contributions of authors are of highest quality and strongly contribute to the increasing international attention in this Research Topic. These results would help plant physiologist in order to improve the understanding of function, regulation and production of relevant metabolites and nutrients that could influence crops yield and help sustainable agriculture challenges.

## Author contributions

RS and JM contributed to the writing of this editorial. All authors revised and improved the final version of the editorial. All authors contributed to the article and approved the submitted version.
